# A High-Dose Shiitake Mushroom Increases Hepatic Accumulation of Triacylglycerol in Rats Fed a High-Fat Diet: Underlying Mechanism

**DOI:** 10.3390/nu6020650

**Published:** 2014-02-12

**Authors:** Dian Handayani, Barbara J. Meyer, Jiezhong Chen, Simon H. J. Brown, Todd W. Mitchell, Xu-Feng Huang

**Affiliations:** 1 Nutrition Department, Faculty of Medicine, University of Brawijaya, Jl. Veteran, Malang 65145, Indonesia; E-Mail: dh753@uowmail.edu.au; 2 School of Health Sciences and Illawarra Health and Medical Research Institute, University of Wollongong, Northfield Avenue, Wollongong, NSW 2522, Australia; E-Mails: todd_mitchell@uow.edu.au (T.W.M.); xhuang@uow.edu.au (X.-F.H.); 3 School of Biomedical Sciences, The University of Queensland, St Lucia, QLD 4072, Australia; E-Mail: j.chen4@uq.edu.au; 4 School of Chemistry and Illawarra Health and Medical Research Institute, University of Wollongong, Wollongong, NSW 2522, Australia; E-Mail: simonb@uow.edu.au

**Keywords:** Shiitake mushroom, beta glucan, high fat diet (HFD), hepatic triacylglycerol

## Abstract

Shiitake mushroom have been shown to have health benefits including lowering plasma lipids and preventing body weight gain. However, their underlying mechanisms are largely unknown. The study aim was to assess the potential underlying mechanism of Shiitake mushrooms in lowering plasma triacylglycerol (TAG) in rats fed a high fat diet (HFD). Forty Wistar rats were divided into control group were given HFD and treatment group were fed HFD, enriched with Shiitake mushroom powder at a low dose (LD-M): 0.7%, medium dose (MD-M): 2%, or high dose (HD-M): 6% (wt:wt) for six weeks. Diets were isocaloric containing ~50% energy from fat. After six weeks’ dietary intervention, the rats were sacrificed, and blood and tissue samples were collected. The HD-M group showed a significantly higher ratio of liver weight to 100 g body weight (*p* < 0.05), a more severe hepatic steatosis marker, such as hepatocyte ballooning (*p* < 0.0001), and more liver triacylglycerol content than LD-M and MD-M (*p* < 0.05). HD-M also showed a significantly decreased ratio of phosphatidylcholine (PC) to phosphatidylethanolamine (PE) compared to HFD (*p* < 0.05), however, there were no differences compared to HD-M and MD-M. Our results also showed a positive association between the dosage, liver TAG, and liver ballooning histology. A negative association was found between the mushroom dosage and the ratio of liver PC to PE. This study showed the mechanism of how high-dose Shiitake mushroom (HD-M) prevents obesity by increasing TAG accumulation in the liver, rather than adipose tissue.

## 1. Introduction

Shiitake mushrooms are a functional food because that contains natural bioactive substances, such as β-glucan and eritadenine. Using a Shiitake mushroom-enriched diet to lower plasma lipid has been widely reported [1,2,3,4]. More recently, the role of Shiitake mushrooms in preventing body weight gain has been reported but its mechanism was largely unknown [5]. The previous study identified that a Shiitake mushroom-enriched diet in rats fed a high fat diet (HFD) significantly lowered plasma triacylglycerol (TAG) and fat deposition by −55% and −35%, respectively, compared to HFD alone [5]. It has also been identified that a high dose mushroom (HD-M) enriched diet significantly increased the ratio of faecal fat to faecal weight by +58% compared to HFD alone [6]. The β-glucan of Shiitake mushrooms was possibly assisting faecal fat exclusion through the effects of β-glucan viscosity [7,8]. From these data it could be hypothesized that Shiitake mushrooms decrease plasma TAG, prevent fat deposition, and prevent body weight gain via faecal fat exclusion and the accumulation of fat in the liver.

Another biological component of Shiitake mushrooms, namely eritadenine, has been reported to have a plasma lipid lowering effect [9,10]. Eritadenine has been reported to be ten times as effective in improving dyslipidaemia as clofibrate [2]. Eritadenine is effective in lowering dyslipidaemia by decreasing the concentration of phosphatidylcholine (PC) and increasing the concentration of phosphatidylethanolamine (PE) in the liver [11,12]. PC is an important phospholipid for lipoprotein assembly and secretion from the liver [13]. Adding eritadenine to the rat diet significantly decreased the level of plasma TAG [9,11] but increased the concentration of TAG in the liver [11]. The accumulation of TAG in the liver contributes to the development of hepatic steatosis [14]. Consistent with this, a Shiitake mushroom enriched diet (5% by weight) was recently reported to induce hepatic steatosis in mice [15].

There is little information regarding the association between the consumption of Shiitake mushrooms as a functional food in obesity prevention and the identification of their underlying mechanism. Thus, the aims of this study were: (1) to determine the liver weight, liver TAG, liver fat histology, liver PE, and liver PC concentrations; (2) to determine the association between Shiitake mushroom dosages and liver fat content; and (3) to determine other potential mechanisms of Shiitake mushrooms in preventing body weight gain in rats fed a HFD.

## 2. Experimental Section

### 2.1. Animals and Diet

All experimental procedures were approved by the Animal Ethics Committee of the University of Wollongong, AE 09/01. Forty Wistar rats were divided randomly into four groups (*n* = 10) and fed 50% HFD modified from standard diet of AIN-93 with an addition of nil, low, medium, or high doses of Shiitake mushroom powder (HFD, 7 g/kg LD-M, 20 g/kg MD-M, or 60 g/kg HD-M, respectively). The dietary intervention was carried out for six weeks as previously described [5]. This study used Shiitake mushroom powder containing 30% β-glucan (w:w) analyzed with a Megazyme β-glucan Kit (K-YBGL 04/2008, Victoria-Australia). It has been shown that Shiitake mushrooms contain eritadenine of approximately 3.86 mg/g of dried Shiitake mushroom [16]. The doses of Shiitake mushroom in this current study contain eritadenine of around 27 mg/kg diet, 77 mg/kg diet, and 232 mg/kg diet in LD-M, MD-M, and HD-M, respectively.

### 2.2. Tissue Collection and Fractionation

At the end of the feeding period, rats were sacrificed via carbon dioxide asphyxiation. The whole liver was quickly removed, weighed, placed in liquid nitrogen, and then stored at −80 °C until it was analyzed.

### 2.3. Liver Crude Fat Weight and Liver TAG Analysis

Hepatic lipids were extracted according to standard procedures [17,18] using ultrapure grade solvent, methanol (MeOH, HPLC grade) from Merck (Darmstadt, Germany), and chloroform (CHCl_3_, HPLC grade) from Honeywell Burdick and Jackson (Muskegon, MI, USA). Analytic grade butylated hydroxytoluene (BHT) was purchased from BDH laboratories (BH15 1TD, Poole, UK). The liver fat was extracted with chloroform/methanol (2:1, by volume) containing 0.01% BHT as an antioxidant. The liver fat extract was dried under nitrogen and the crude fat was weighed. After redissolving in *n*-hexane, the liver TAG was analyzed [8,19] using the Konelab^®^ 20XT automatic analyzer with the Infinity™ reagent from Thermo Fisher Scientific (Auburn, NSW, Australia).

### 2.4. Liver Histology

We examined the liver lipid accumulation using Oil Red O-stained as described previously [8]. Briefly, frozen rat livers were sliced in 10 μm sections using a cryostat (LEICA, Wetzlar, Germany) and fixed with ice cold 10% formaline. The livers were air dried for 60 min and rinsed immediately in distilled water, 3 times. After air drying for approximately 5 min, they were placed in an absolute propylene glycol solution for 5 min and stained with pre-warmed Oil Red O solution (Oil Red O-SIGMA, St Louis, MO, USA) for 10 min at 60 °C. The slices were differentiated in 85% propylene glycol solution for 5 min, then stained in Mayer’s hematoxylin and eosin (Sigma Chemical, St Louis, MO, USA) for 30 s. The slices were washed thoroughly in running tap water for 3 min. The slices were mounted onto slides using glycerine jelly and then covered with cover slips.

Histological images of Oil Red O-stained (ORO) liver sections were observed at 10 × 0.30 with a 0.17/A Fluotar, a 10 × 0.30 Leitz DMRB microscope and captured with a Leica CCD digital camera, using a standard exposure for all photographs.

The histological features were identified using steatosis and ballooning classification. They were scored using a previous method [14]. The steatosis grades were grouped as: <5%, scored 0; ≥5%–33% scored 1; >33%–66%, scored 2; >66%, scored 3. The ballooning classifications were grouped as: 0 if ballooning was not observed; 1 if only a few balloon cells were observed; and 2 if there was prominent cell ballooning.

### 2.5. Liver Phospholipids Analysis

The liver was homogenized in four volumes (v:wt) of cold methanol/chloroform (1:2, by volume) containing 0.01% BHT. Dinonadecanoyl phosphatidylcholine (PC 19:0/19:0) and diheptadecanoyl phosphatidylethanolamine (PE 17:0/17:0) were added to the homogenate as internal standards with final concentrations of 1 and 0.75 µmol/g of tissue respectively. The total lipids were extracted as described previously [20] and stored at −80 °C until analysis. The liver extracts were analyzed on a QTRAP 5500 mass spectrometer (AB Sciex, Concord, Vaughan, Canada) combined with a Nanomate Triversa robotic nanospray source (Advion Biosciences, Ithaca, NY, USA). All samples were infused with a spray gas pressure of 0.4 psi and voltage of 1.2 kV. Lipid extracts were diluted 100-fold into MeOH/CHCl_3_ (2:1, by volume) containing 7.5 mM ammonium acetate. PC was identified by precursor ion scanning for the *m/z* 184.1 product ion in the positive mode. PE was identified by neutral loss scanning for a loss of 141 Da in the positive mode. Collision energy was set at 55 and 30 eV respectively for precursor ion and neutral loss experiments and 400 scans were summed for each experiment. Data were analyzed with LipidView [21] including de-isotoping, smoothing and isotope correction. Lipid concentrations were calculated using LipidView by comparison with internal standards. Lipid concentrations were then exported to Excel [22]. The ratio of PC to PE was calculated as PC concentration per PE concentration (mol/mol).

### 2.6. Statistical Analysis

Data were presented as mean ± standard error of the mean (SEM). TAG liver data were transformed to square root values to achieve normality before significance testing. One-way analysis of variance (ANOVA) was used, followed by a *post hoc* Tukey-Kramer significant differences test for multiple comparisons among the groups. Differences were considered significant when *p* < 0.05. A simple association between two variables was calculated using Pearson’s correlation coefficient. All statistical analysis was performed using SPSS software (version 17.0, SPSS Inc., Chicago, IL, USA).

## 3. Results

### 3.1. Liver Weight

The liver weights were not significantly different between the four diet groups (Table 1). However, the liver weight per 100 g body weight was significantly different. We found that HD-M showed a significantly higher liver weight, per 100 g body weight, compared to LD-M (Table 1, +14%, *p* = 0.013) and MD-M (Table 1, +17%, *p* = 0.015), respectively. However, there was no significant difference in liver weight per 100 g body weight in HD-M compared to that in the HFD group.

**Table 1:** Body weight, liver weight, liver triacylglycerol (TAG) and liver phospholipid concentration in rats fed a high fat diet (HFD) enriched with Shiitake mushrooms.

Parameter ^1^	HFD	LD-M	MD-M	HD-M	ANOVA
**Body Weight**	479 ± 7	512 ± 23	516 ± 26	480 ± 20	NS
**Liver**					
Liver weight (g)	19 ± 1	18 ± 1	17 ± 1	20 ± 1	NS
Liver/100 g body weight	3.9 ± 0.2 ^a,b^	3.6 ± 0.2 ^b^	3.5 ± 0.1 ^b^	4.1 ± 0.1 ^a^	0.008
Liver TAG (μmol/g tissue)	45 ± 11 ^a,b^	21 ± 2 ^a^	19 ± 4 ^a^	69 ± 14 ^b^	0.001
**Liver Phospholipid**					
PC (nmol/mg)	34 ± 1	38 ± 2	39 ± 2	34 ± 0.2	NS
PE (nmol/mg)	9.7 ± 0.4 ^a^	10.6 ± 0.6 ^a,b^	11.9 ± 0.4 ^b^	11.8 ± 0.6 ^a,b^	0.037
Ratio PC/PE (mol/mol)	3.5 ± 0.1 ^b^	3.5 ± 0.1 ^b^	3.3 ± 0.1 ^a,b^	2.9 ± 0.1 ^a^	0.001

TAG: Triacylglycerol; PC: Phosphatidylcholine; PE: Phosphatidylethanolamine. ^1^ Values are mean ± SEM. ^a,b^: Within a row, the different superscripts are mean significantly different, *p* < 0.05. HFD, high fat diet; LD-M, low dose mushroom in HFD; MD-M, medium dose mushroom in HFD; HD-M, high dose mushroom in HFD.

### 3.2. Liver Total Fat Content

**Figure 1:**
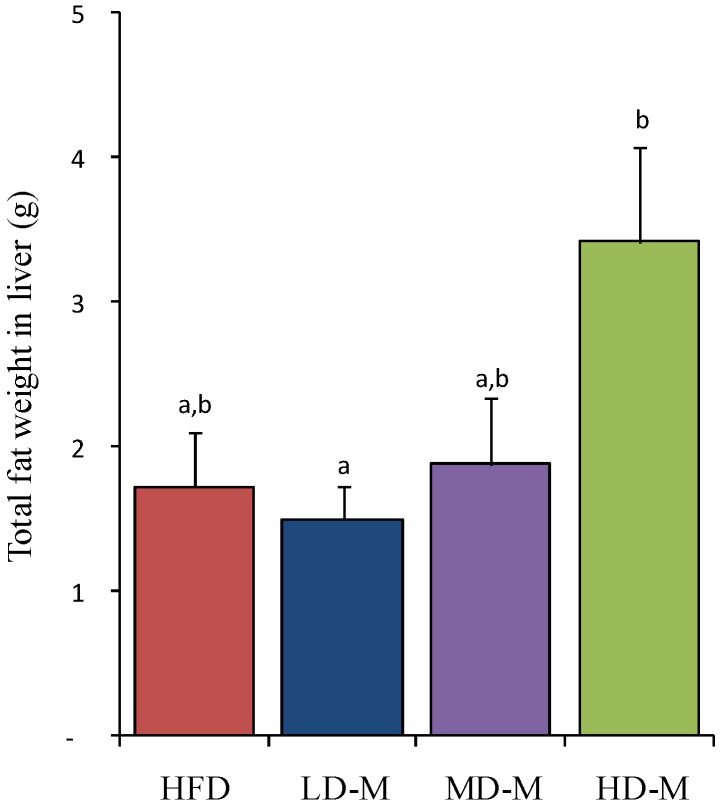
The total fat in the liver was measured after 6 weeks dietary treatments in rats. Bars represent mean ± SEM. ^a,b^ Means not sharing a common letter are significantly different among groups at *p* = 0.024. HFD, high fat diet; LD-M, low dose mushroom in HFD; MD-M, medium dose mushroom in HFD; HD-M, high dose mushroom in HFD.

The liver total fat was significantly different among the four diet groups (Figure 1; *p* = 0.024). We found significantly higher levels of liver total fat mass in HD-M compared to LD-M (Figure 1, +128%; *p* = 0.033) and a trend to higher levels compared to HFD (Figure 1, +98%; *p* = 0.052), however HD-M was not different compared to MD-M.

### 3.3. Liver Histology

The HFD and mushroom enriched diets were not significantly different in hepatic steatosis using the histological scoring system (Table 2; *p* = 0.472). However, the assessment of the ballooning hepatosteatosis scoring revealed that the HFD and mushroom enriched diets were significantly different (Table 2; *p* < 0.001).

**Table 2:** Assessment of hepatosteatosis by histological scoring system on rats fed HFD enriched with Shiitake mushrooms.

Parameter ^1^	HFD	LD-M	MD-M	HD-M	ANOVA
**Liver steatosis**	2.70 ± 0.20	2.83 ± 0.17	2.67 ± 0.21	3.00 ± 0.09	NS
**Hepatic ballooning**	0.0 ± 0.0 ^a^	0.5 ± 0.2 ^a^	0.3 ± 0.2 ^a^	2.0 ± 0.0 ^b^	0.000

Liver steatosis: 0: <5%; 1: 5%–33%; 2: >33%–66%; 3: >66%. Hepatocyte ballooning: 0, none; 1, few ballooning cells; 2, many cells/prominent ballooning. ^1^ Values are mean ± SEM. ^a,b^: Within a row, the different superscript are mean significantly different, *p* < 0.05*.* HFD, high fat diet; LD-M, low dose mushroom in HFD; MD-M, medium dose mushroom in HFD; HD-M, high dose mushroom in HFD.

**Figure 2:**
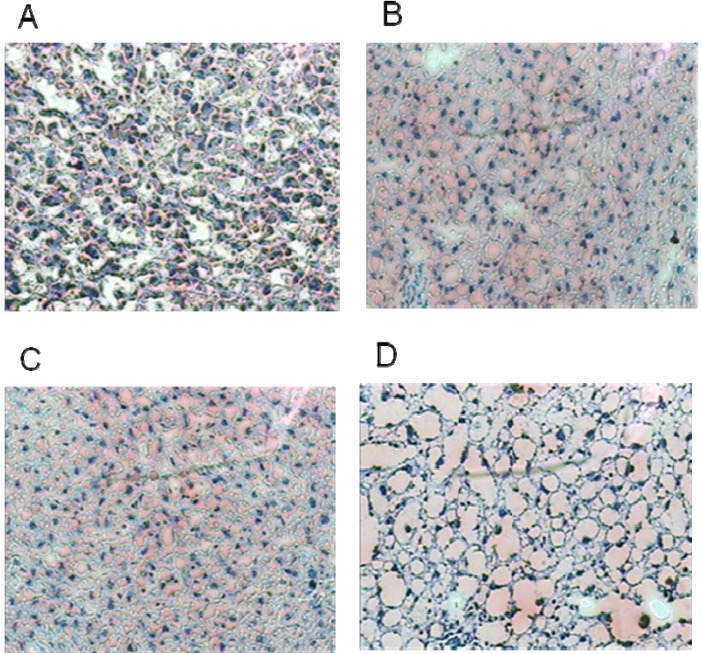
The effect of the Shiitake mushroom enriched diet on lipid droplet deposition of hepatic tissue by Oil Red O staining on rats fed HFD. (**A**) HFD—control diet; (**B**) low dose mushroom; (**C**) medium dose mushroom; (**D**) high dose mushroom.

This study found HD-M had significantly more hepatic cell ballooning than HFD, LD-M, and MD-M (Table 2; all *p* < 0.001). There was no significant difference in hepatic cell ballooning in HFD compared to that in LD-M and MD-M (Figure 2).

### 3.4. Liver Triacylglycerol (TAG)

The liver TAG level was significantly different among the four diet groups (Table 1, *p* = 0.001). We found significantly higher levels of liver TAG in HD-M compared to LD-M (Table 1, +229%, *p* = 0.007) and MD-M (+257%, *p* = 0.001). HD-M was not significantly different compared to HFD.

### 3.5. Liver Phosphatidylcholine (PC) and Phosphatidylethanolamine (PE)

The concentration of PC in the liver did not differ among the four groups. The concentration of PE was significantly increased in MD-M compared to HFD (Table 1; 22%; *p* = 0.050). HD-M tended to have a higher PE concentration than that in HFD (Table 1; 21%; *p* = 0.053). However, the concentration of PE in LD-M was not significantly different compared to HFD. The liver PC:PE ratio was significantly different among the groups (Table 1; *p* = 0.001). More specifically, we found a statistical difference in the PC:PE ratio between HD-M compared to HFD (−16%; *p* = 0.001) and to LD-M (−15%; *p* = 0.005). However, we did not find any differences in the PC to PE ratio in rats fed HD-M and MD-M.

### 3.6. Correlation between the Dosages of Shiitake Mushrooms on Liver Weight, Liver Histology, Liver TAG, and Liver PC:PE Ratio

**Figure 3:**
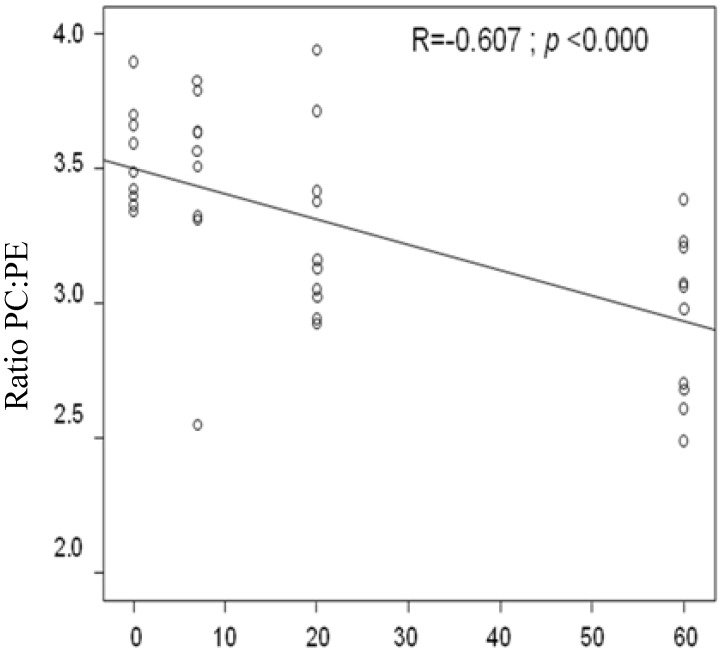
A significant correlation was found between dosage and the ratio of PC to PE; PC: phosphatidylcholine; PE: phosphatidylethanolamine.

We found that the amount of Shiitake mushrooms added to the HFD was positively correlated with the liver TAG (*R* = 0.406; *p* = 0.017) and hepatic cell ballooning histology (*R* = 0.878; *p* < 0.000). On the other hand, the dosages of Shiitake mushrooms showed a negative association with the ratio of PC to PE (Figure 3; *R* = −0.607; *p* < 0.0000). There was no statistical association between the amount of Shiitake mushroom and the liver weight (data not shown).

Shiitake mushroom powder (g/kg diet).

## 4. Discussion

This study identified the effects of a Shiitake mushroom enriched diet on liver/body weight, liver TAG, liver steatosis, and the ratio of PC:PE in the livers of rats fed a HFD.

We found no significant differences in liver weight. However, HD-M showed a significantly higher liver weight, per 100 g body weight, compared to LD-M and MD-M (*p* = 0.008). In general, studies on mushroom enriched diets have shown decreased liver weight for hamsters [23] and rats [24,25,26,27]. However, these results depended on the variety of mushroom and the nutritional composition of the diet. For example, additional *Agaricus bisporus* or white button mushroom [26], straw mushroom [23], and Maitake mushroom [28] were not reported to increase liver weight. An additional 50 mg/kg diet of eritadenine derived from Shiitake mushrooms was reported to increase liver weight significantly (+68%) in rats fed a choline deficiency diet for two weeks compared to the control group which were fed an eritadenine enriched diet with added choline chloride (8 g/kg) [29]. Another study [11] suggests that increasing liver weight is a specific effect of Shiitake but not other mushrooms. Furthermore, this effect is reversed when Shiitake mushrooms are consumed concurrently with sufficient choline [11].

The liver weight expressed per body weight is higher in HD-M compared to LD-M and MD-M (Table 1). This is a consequence of reduced visceral fat mass [30] and body weight in the HD-M group compared to the LD-M and MD-M groups, rather than to differences in liver weight between these three groups. This is in contrast to the studies by Sugiyama *et al*. [11,29]. The difference in liver weight between our study and the Sugiyama study is possibly due to the additional choline chloride in the diet of the rats in the Sugiyama study.

Recently, Shiitake mushrooms were reported to increase vacuolated hepatocytes and hepatic steatosis suggesting fatty liver (*p* < 0.05) in mice fed a normal diet enriched with Shiitake mushrooms for six weeks [15]. Again, Shiitake mushrooms produced significant hepatic steatosis in mice fed a standard diet (AIN-93) compared to mice fed a white button mushroom-enriched diet [15]. In the current study, we found that the HFD induced a significantly higher increase in fat droplets than the LFD (data not shown), highlighting the effect of a mushroom enriched HFD in developing hepatic steatosis. Moreover, the ballooning of hepatocytes was shown to increase with increasing mushroom doses. This is consistent with the studies conducted on mice by Chandra *et al*. [15].

As can be seen from this study, the increase in liver weight/100 g body weight in HD-M was higher than in the HFD and the MD-M and LD-M diet groups. There was a positive correlation between the accumulation of fat in the liver and the dose of Shiitake mushroom powder. The increased liver fat accumulation is an adverse effect from HD-M and is called hepatic steatosis. Previous studies carried out to test the effect of enriched mushroom diets on liver TAG have yielded varying results. For example, in an *Agaricus bisporus* powder enriched diet, rats fed a hypercholesterolaemic diet had significantly lower TAG levels [26]. The addition of 20% (wt:wt) Maitake mushroom powder in a hypercholesterolaemic diet also significantly lowered plasma TAG levels in Sprague-Dawley rats [27].

The study by Sugiyama *et al*. (1997) showed that the increase in total liver fat and liver TAG was followed by increased liver weight [24]. This result is consistent with a previous study that reported on a dietary intervention with eritadenine derived Shiitake mushrooms in choline deficiency rats increase liver TAG and liver weight by 68% and >700%, respectively [11]. Liver TAG is derived from dietary fat that is transported from the intestine, fatty acids from the unesterified fatty acids (FFA) pool, and fatty acids from *de novo* lipogenesis [31]. Normally, TAG is not stored in the liver and is released as the lipoprotein very low-density lipoprotein (VLDL). From the current and previous studies it can be seen that the effects of mushroom supplementation on liver weight, liver TAG levels and fat droplets in the liver depends on the variety of mushroom. The mushroom with eritadenine content will induce liver TAG accumulation when choline chloride is insufficient in the diet. Thus, the presence of eritadenine alters the homeostasis of phospholipid synthesis and storage. The imbalance of phospholipid levels in the liver will affect the level of plasma TAG.

In the current study, we have identified the effect of a Shiitake mushroom enriched diet on the change in the concentration of liver phospholipids, especially the concentration of phosphatidylcholine (PC) and phosphatidylethanolamine (PE). The eritadenine from Shiitake mushrooms has been reported as having the potential to lower lipid levels by changing the number and species of phospholipids in the liver [24]. In general, enriched eritadenine derived from Shiitake mushrooms will induce PC deficiency [10,29]. For example, studies on additional eritadenine derived from Shiitake have reported significantly decreased PC (−42%) if the rats were fed a choline deficient diet [11]. However, additional eritadenine derived from Shiitake mushrooms did not significantly increase liver total fat, liver TAG and liver weight when the eritadenine derived from mushrooms was given concurrently with choline supplementation [11]. The PC deficiency could be prevented by the addition of choline chloride [10,24,29]. Sugiyama *et al*. [11] reported that an additional 8 g choline chloride/kg in the rat diet concurrently with eritadenine derived from Shiitake mushrooms were important to prevent increased liver TAG, and that the additional diet was sufficient to prevent increased liver TAG and liver weight.

The current study had no added choline chloride as a dietary supplement. The source of choline in this study was synthetic vitamins that were added as vitamin B12 and folic acid to the diet, and from vitamin B12 and folic acid in Shiitake mushrooms. The average vitamin B12 and folic acid content in HD-M, MD-M and L-DM was ~40 mg/kg. Therefore, the insufficient choline chloride in the Shiitake mushroom enriched diet (HD-M) explains the hepatosteatosis that was consistent with other studies showing that it promotes hepatosteatosis [11].

Normal levels of PC are necessary for normal secretion of very low density lipoprotein (VLDL) and to maintain homeostasis of TAG lipoprotein release from the liver [9,10,12,29,32]. PC can be produced from the synthesis of PE via phosphatidylethanolamine *N*-methyltransferase (PEMT) and cytidine diphosphate-choline (CDP-choline) pathways [10,33]. The CDP-choline pathway depends on dietary choline while the PEMT pathway for PC biosynthesis does not. The PC biosynthesis in the PE *N-*methylation pathway only produces 30% of the total PC in the liver, therefore, the greatest PC biosynthesis occurs via the CDP-choline pathway [34]. In general, eritadenine from a Shiitake mushroom enriched diet increases the concentration of PE via the CDP ethanolamine pathways [10,11,35]. Our study has also shown that diets enriched with Shiitake mushrooms significantly increased PE compared to HFD. Indirectly, eritadenine prevented the production of PC from the PE *N*-methylation pathway, although it also increased PEMT activity [11]. However, even though eritadenine increased PEMT activity, increasing PEMT activity did not automatically increase PC from the PEMT methylation pathway because the PE-N methylation reaction not only needs enzyme mass but also a substrate such as *S*-adenocyl methionine (AdoMet). Eritadenine was reported to decrease PC from the PEMT pathway by inhibiting *S*-adenocyl hydrolyse [29,36]. The inhibition of *S*-adenocyl hydrolyse induces an increase of *S*-adenocyl homocysteine (AdoHcy) and thus decreases the ratio of AdoMet to AdoHcy. The decrease in this ratio inhibits PE-N methyltransferase to reduce the synthesis of PC from PE; therefore it will decrease PC production in the liver [11].

The ratio of PC:PE could also be used as the marker of disturbance of hepatic membrane permeability [37]. Study from Li and Agellon [37] reported that a decreased ratio of PC:PE promoted an increased hepatic steatosis in *Pemt^−^*^/−^ gene disrupted mice fed a choline deficiency diet. This study found that the increasing of mushroom powder doses in food decrease the ratio PC:PE, it means HD-M has the lowest ratio PC:PE among the groups. It was consistent with other studies from Sugiyama, Akachi, *et al*., 1995 [11], and Sugiyama, Yamakawa, *et al*., 1997 [24]. The lowering of ratio PC:PE affect on increasing of hepatic membrane permeability. It will induce ballooning of hepatocytes as a result of cell damage, which is typical of hepatic steatosis. The positive association of liver TAG and liver ballooning with mushroom dosage showed that the HD-M of Shiitake mushrooms had a high enough level of eritadenine to decrease the ratio of PC:PE in the current study. Our finding that a decrease in this ratio in a choline deficient diet will induce lowered plasma TAG levels and cause liver fat accumulation is consistent with previous studies [12,38].

For that reason, fatty liver in rats fed an eritadenine-enriched diet could be effectively prevented by a choline chloride diet (8 g/kg) [11]. Adequate choline in a Shiitake mushroom enriched diet would not impair the release of VLDL, as the eritadenine from Shiitake mushrooms neither increased the TAG concentration of the liver nor decreased the plasma concentration [11]. On the other hand, hepatic steatosis has been reported to be reversible when mice are withdrawn from Shiitake mushroom consumption [15]. Therefore, consuming Shiitake mushrooms for combating obesity could still be considered, as long as they are consumed at a safe dose and as part of a diet containing sufficient choline chloride over a short period.

## 5. Conclusions

In conclusion, we have shown that the underlying mechanisms of HD-M diet to prevent body weight gain and reduction in plasma TAG levels were due to fat (TAG) accumulation in the liver resulting in severe hepatic steatosis. Whether or not this unwanted side effect can be prevented by the addition of choline to the diet requires further investigation.
